# Editorial: Microbial safety of animal-based food products

**DOI:** 10.3389/fnut.2025.1561962

**Published:** 2025-02-06

**Authors:** María J. Andrade, Micaela Álvarez, Paula Rodrigues

**Affiliations:** ^1^Higiene y Seguridad Alimentaria, Instituto Universitario de Investigación de la Carne y Productos Cárnicos, Facultad de Veterinaria, Universidad de Extremadura, Cáceres, Spain; ^2^Sección Departamental de Nutrición y Ciencia de los Alimentos, Facultad de Veterinaria, Complutense University of Madrid, Madrid, Spain; ^3^Centro de Investigação de Montanha (CIMO), Instituto Politécnico de Bragança, Campus de Santa Apolónia, Bragança, Portugal

**Keywords:** pathogens, foodborne diseases, predictive microbiology, biopreservation, organic food

## Introduction

The consumption of food of animal origin has been linked to the development of a wide range of illnesses. For example, diet plays a crucial role in neurodegeneration, cardiovascular diseases as well as carcinogenic processes. Specific foods and nutrients of animal origin affect the emergence and progression of certain diseases such as the exposure to saturated fats or carcinogenic additives.

Moreover, many pathogenic microorganisms can be found in animal-derived food, such as meat, fish, eggs, milk and honey, being crucial sources of human diseases and even deaths. Their presence can be due to contamination in the production environment and/or during the processing of derived products because of human handling and environmental conditions. Thus, their control throughout the entire food chain is crucial.

Food producers specializing in food products of animal origin need to implement effective strategies to avoid the presence of pathogenic microorganisms and microbial toxins and thus protect the consumers' health. Basic actions are proven effective in reducing this hazard, but new insights are necessary due to some emerging factors, such as changing lifestyles, climate change, and new export chains. Furthermore, consumers are also increasingly interested in environmentally sustainable food production methods, including organic food and natural antimicrobial additives. In addition, it is crucial to obtain more details about the relationships between the consumption of food of animal origin and the development of some illnesses like colorectal cancer.

This Research Topic, aims to compile both research articles and reviews in the field of Microbial Safety of Animal Products. Special attention was given to works focused on innovative approaches to guarantee the control of biological hazards in this kind of food, as well as on the current challenges from the food safety point of view. The collected research articles provide perspectives on the epidemiological characteristics and etiology of such illnesses related to food of animal origin consumption as well as natural strategies to be used as antimicrobials and novel techniques to detect foodborne pathogen contamination.

## New strategies to detect and avoid undesirable microorganisms in food of animal origin

One of the first studies on the Research Topic conducted by Bai et al., is focused on the rapid and accurate detection of foodborne pathogens on mutton by using short-wave infrared hyperspectral imaging (SWIR-HSI). The combination of SWIR-HSI with traditional machine learning and deep learning methods could effectively detect the contamination and identify the pathogens in muttons. The performance of deep learning model was better than that of machine learning. This study highlights the importance of establishing rapid and robust techniques for foodborne pathogens detection adapted to the food characteristics.

On the other hand, essential oils are known for their strong antibacterial properties making them a natural strategy instead of chemical additives. However, their chemical instability and impact on meat proteins limit their application. To avoid these limitations, Wu K. et al. compare the activity of cinnamon essential oil (CEO) loaded in a liposome or an emulsion on the proteolysis of minced pork and the advantages of each delivery system in preventing the development of undesirable microorganisms. The results indicate that CEO-liposome was an effective preservative for minced pork. Minced pork treated with CEO-liposomes exhibited lower microorganism levels than CEO-emulsions, providing better protection. Additionally, CEO-liposome produced lower amounts of bitter amino acids and biogenic amines. This article fosters the use of natural bioactive strategies to prevent unwanted microorganisms without neglecting the sensorial quality of the meat.

## Risk factors involving food of animal origin in the development of illnesses

In relation to food safety, consumption of foods of animal origin should not be a risk factor for those suffering from any clinical syndrome. However, the article submitted by Wu Y. et al., describes, for the first time, the association between the consumption of crayfish in Wuhan (China) and the increase of rhabdomyolysis that causes muscle pain, weakness, and chest distress. The symptoms appeared within 24 h after the consumption of cooked crayfish and other aquatic products. This suggests that the intake of crayfish in large quantities may contain certain toxins that trigger the onset of rhabdomyolysis. Therefore, the article supports the need to study and control not only the microbial pathogens but also the possible toxins present in food.

Finally, the study of Kim et al. is focused on the association between fish and meat intake and the development of colorectal adenoma in the Korean population. No clear association was found between the intake of total fish, meat, red meat, chicken or processed meat and the prevalence of colorectal cancer taking into account genetic and lifestyle factors. However, they observed a significant inverse relation between high fish intake and the high risk of developing adenoma. The study provides new information for nutritional advice and improvement of healthy dietary and lifestyle practices for the primary prevention of colorectal cancer.

## Summary

The studies gathered in this Research Topic focus on several key areas to ensure the safety of animal food products. Firstly, rapid pathogen detection is being improved through technologies like SWIR-HSI combined with advanced machine-learning techniques. Secondly, the use of natural antimicrobials, such as essential oils delivered via liposomes, is being explored to combat microbial development while minimizing negative impacts on food quality. Thirdly, investigations have revealed a link between high crayfish consumption and rhabdomyolysis, emphasizing the need to control the toxins in the food chain. Finally, studies on the relationship between diet and colorectal cancer have suggested a potential inverse association between high fish intake and risk of developing colorectal adenoma, suggesting a possible preventative role for fish consumption. These combined efforts aim to enhance the safety and quality of animal-derived foods through advanced detection methods, natural preservation strategies, and a broader understanding of foodborne threats.

